# Free fatty acids esterification catalyzed by acid Faujasite type zeolite

**DOI:** 10.1039/c8ra10248a

**Published:** 2019-02-08

**Authors:** Daniel Marcos Dal Pozzo, José Airton Azevedo dos Santos, Edward Seabra Júnior, Reginaldo Ferreira Santos, Armin Feiden, Samuel Nelson Melegari de Souza, Ismael Burgardt

**Affiliations:** Federal Technological University of Parana (UTFPR) Avenida Brasil 4232 85884-000 Medianeira Brazil danielpozzo@utfpr.edu.br; Engineering of Energy in Agriculture, State University of West Parana, Rua Universitária 2069 85819-110 Cascavel Brazil

## Abstract

The catalytic activity of the protonated form of H-Y(80) zeolite (Faujasite with high Si/Al ratio) was evaluated as an acid catalyst in the esterification step pre-treatment of FFA by means of the esterification reaction of oleic acid with methanol in soybean oil. The zeolite structure was characterized by XRD and FTIR. Textural characterization was carried out by N_2_ physisorption. The thermal stability was evaluated by TG-DTA and the acidity measured by NH_3_-TPD and Pyridine-FTIR. The limitations of the use of this zeolite in a pre-treatment for biodiesel production was investigated through oleic acid esterification in soybean oil, as a model reaction, performed with different temperatures, catalyst amounts and molar ratios. The results showed that the amount of remaining FFA decreased to values well below the initial amount. Under the optimal reactional conditions, conversions to methyl esters above 95% were achieved. Results support that such reactions can be performed under H-Y(80) zeolite catalysis and can be applied in a pre-treatment esterification of feedstocks with high contents of FFA. Catalyst reuse is feasible due to its easy separation from reaction products allowing new reaction cycles, as well as the application of the H-Y(80) zeolite in biodiesel production.

## Abstract 2

FFA esterification pre-treatment catalyzed by a Faujasite type zeolite.

## Graphical abstract

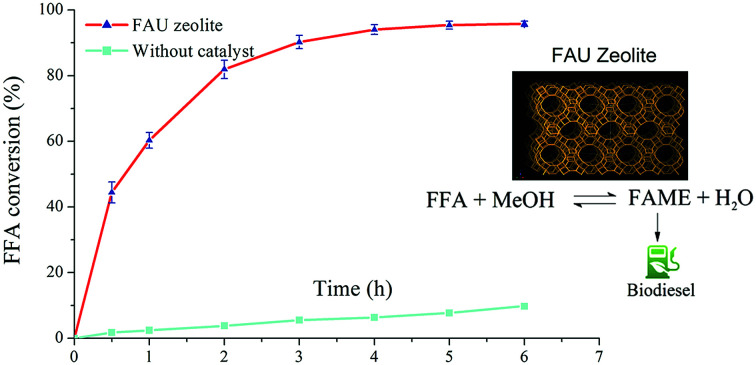

## 1. Introduction

Energy is the key determinant for sustainable development worldwide.^1^ Global primary energy consumption has increased by about two times in the last 40 years.^2^ Nowadays, approximately 80% of global primary energy demand is supported by fossil fuels, contributing to a range of environmental and human health concerns.^3,4^ The growing global energy demand and consequences related to the use of fossil fuels have led to the search for renewable energy sources.^5^ Within this context, the replacement of fossil fuels with biofuels is interesting because of its environmental benefits, since they can be obtained from renewable sources, and contributes to making the global energy matrix less dependent on fossil fuels.^6^

The use of residual oils (raw materials) is an interesting alternative for biodiesel production due to the lower cost of the feedstocks and possibility of using tailings of a certain process as feedstocks for the production of biodiesel collaborating for environmental sustainability.^7–10^

Homogeneous base catalyzed transesterification reaction is a commercial technology to produce biodiesel, but it is not applicable to process residual oils.^11^ Unfortunately, waste cooking oils present a high content of water and FFA (Free Fatty Acids), which is a problem for the conventional process that occurs under alkaline transesterification.^12^ The presence of water and FFA in the reaction medium results in the formation of soap, decreases the yield of the reaction, cause catalysts deactivation, and additionally, increases the generation of byproducts on the purification and washing steps.^13,14^

There are two possible solutions to this problem. The first one is the use of metal based catalyst to remove oxygen, in the presence or absence of H_2_, in the form of CO, CO_2_ or H_2_O by deoxygenation reaction, transforming the feedstock into liquid hydrocarbons which are ideal replacements for conventional fossil fuels.^15–17^ Deoxygenation of fatty acids and triglycerides has been studied with various metal catalysts,^18–24^ but this process becomes less interesting when conducted in the presence of H_2_, due to its higher cost, difficulties of storage and transportation.^15,21^

The other method is the use of acid base catalysts instead of base catalysts. Acid homogeneous catalysts received wide acceptability because of their fast reaction rates, however, postproduction costs incurred from aqueous quenching, wastewater and loss of catalysts led to the search for alternatives.^25^ In this context, the application solid acid catalysts is an alternative process due to the easy separation of the catalyst from the reaction medium at the end of the reaction and possibility of reuse in new reaction cycles.^26–28^

Among the heterogeneous catalysts has been studied for esterification such as Amberlyst 15,^29^ Amberlyst 46,^30^ sulfonated carbon,^31^ sulfonic acid-funcionalized pyrazinium^32^ niobic acid,^33^ tungsten oxide supported,^34^ sulfated zirconia^35^ and zeolites.^36^

Zeolites are highly symmetrical crystalline materials, widely used as catalysts in the petrochemical industry, as well as being used in other applications as adsorbents and ion exchangers in wastewater treatment.^37^ The zeolites are composed by silicon tetrahedra [SiO_4_] and aluminum [AlO_4_] joined at the vertex by oxygen atoms and arranged in a three dimensional structure with pore structure of molecular dimensions, with high surface area, thermal and chemical stability.^38,39^

Among the different zeolites framework topologies, the Faujasite type zeolite (FAU zeolite) has a twelve member ring with a pore aperture from 7.4 Å,^40^ that can be used for the esterification reaction, but it catalytic activity is dependent on a number of parameters, such as porosity, acidity and its hydrophobic character.^41^

In this context, Doyle *et al.*^42,43^ investigated the esterification of oleic acid over H-Y zeolite obtained from shale rock and reported good conversions. Prinsen *et al.*^41^ reported that the zeolite FAU (with Si/Al = 15) is as a sustainable catalyst for esterification, because it can catalyze the reaction faster and exhibit catalytic activity for several consecutive reaction cycles.^41^ Alismaeel *et al.*^44^ obtained a FAU-type zeolite with a high catalytic activity for esterification of oleic acid and concluded that the zeolites are suitable heterogeneous catalysts for the biodiesel preparation.

For the biodiesel production, low grade feedstocks with high acidity can also be subjected to a pre-treatment of esterification of the FFA so that the process can be continued after by means of the alkaline transesterification.^13,27,41^

The aim of this study was to evaluate the catalytic activity of the protonated form of Y zeolite (H-Y) applied as catalyst in the FFA esterification pre-treatment, by means of the esterification reaction of oleic acid with methanol in soybean oil, aiming to analyze the limitations of the use of this zeolite in a pre-treatment for the production of biodiesel, in order to give information that show innovative alternatives to the conventional biodiesel production process.

## 2. Experimental

### 2.1 Materials

FAU-type zeolite (H-Y with SiO_2_/Al_2_O_3_ = 80) in the proton form (H-Y), denoted H-Y(80), was purchased from Zeolyst International – USA. This zeolite was employed in the reactions in its proton form (H-Y). Before the reactions carried out, the catalyst was submitted to a dehydration treatment at 105 °C for 1 h. Its physicochemical properties, as acquired, are shown in Table 1.

**Table 1:** Physicochemical properties of the catalysts^45^

Catalyst	Molar ratio SiO_2_/AlO_3_	Pore size^46^ (Å)	Surface area (m^2^ g^−1^)
H-Y(80)	80	[111] 7.4 × 7.4 12-MR	780

Methanol (MeOH) and Oleic Acid (OA) were purchased from Sigma-Aldrich (analytical or higher grade) and were used without further purification.

### 2.2 Catalyst characterization

The elemental chemical composition of the H-Y(80) was determined by Energy Dispersive X-ray Fluorescence (ED-XRF), in a Shimadzu X-ray Fluorescence Spectrometer, model: EDX-720.

The zeolite thermal stability was evaluated by TG-DTA analysis in a in the PerkinElmer STA 6000 Thermo-Analyzer. A sample of 10 mg was subjected to a temperature increase of 40 to 600 °C, under N_2_ flow of 20 L min^−1^ using a heating rate of 10 °C min^−1^.

The zeolite structure was characterized by FTIR and XRD. For the FTIR experiments, the zeolite sample was dispersed in KBr (dried at 105 °C for 3 h) in a 13 mm diameter disc. The FTIR spectra were acquired in the range of 4000–500 cm^−1^, averaging 16 scans, at a resolution of 2 cm^−1^.

X-ray powder diffraction (XRD) patterns were acquired with a PANalytical Empyrean X-ray Powder diffractometer using Cu Kα radiation (*λ* = 1.5405 Å), the data was recorded from 4 to 50° 2-theta with step size 0.032° operating at 40 kV and 30 mA with fixed 1/4′′ anti-scatter slit.

TPD-NH_3_ experiments were obtained using a bench flow system (SAMP-3) with thermal conductivity detector. Prior to each TPD run, 0.2 g of the H-Y(80) was pre-treated at 250 °C for 30 min under pure He (30 mL min^−1^, rate 10 °C min^−1^). The sample was then cooled to 100 °C and a flow of 30 mL min^−1^ of 5% NH_3_/He was used to adsorb ammonia for 30 min. The sample was purged with pure He (30 mL min^−1^, 100 °C) for 30 min to remove excess ammonia before obtaining the TPD profile, where the temperature was ramped up to 800 °C at 10 °C min^−1^ with 30 mL min^−1^ of He.

Acidity measurements also were performed according the Pyridine Fourier Transform Infrared Spectroscopy (Pyridine-FTIR) adsorption method proposed by Zanatta and coworkers, the experimental procedures are described elsewhere.^47^ For this procedure, the H-Y(80) zeolite was submitted to pyridine adsorption evaluated by FTIR, according to the vibrational molecular modes from 4000 cm^−1^ to 500 cm^−1^ region, aiming to observe the presence of the acidic sites in its structure. FTIR spectra were acquired using the same procedure already described.

N_2_ physisorption isotherms were obtained at −196 °C in a Quantachrome Nova 2200e. Prior to the analysis, the sample was pretreated at 300 °C under vacuum for 4 h. The surface area was calculated by BET and DFT method, and the volume, pore distribution and pore size were obtained by the BJH method.

### 2.3 FFA esterification pre-treatment

The catalytic activity of the H-Y(80) zeolite for the esterification pre-treatment was evaluated using as model reaction the esterification of oleic acid with methanol in soybean oil.

A solution of oleic acid with soybean oil containing 10% oleic acid in mass was prepared, in order to simulate low grade feedstock with high acidity (high content of FFA). The temperature influence on the conversion of fatty acids at conditions of 55 °C and 68 °C was evaluated, as well as the alcohol excess effect (molar ratios of 1 : 3, 1 : 6 and 1 : 9), besides the amount of catalyst, where 3 and 5% of zeolite was used in relation to the mass of the solution. An uncatalyzed reaction was performed at 68 °C and molar ratio of 1 : 6.

At the specified times (0.5, 1, 2, 3, 4, 5 and 6 h), an aliquot of sample was collected and centrifuged at 3800 rpm for 4 min to separate the zeolite from the organic phase. In the sequence, the supernatant was collected for the quantification of the conversion of the FFA initially present in the reaction medium.

The quantification of FFA conversion was performed by titration with 0.1 M solution of KOH and phenolphthalein as indicator from the relationship between the initial acidity of the reaction medium and the acidity of the sample in the specified time interval. The experimental procedures are already described elsewhere^43,48^ so will not be repeated here. All samples were analyzed in triplicate.

## 3. Results and discussion

### 3.1 Catalyst characterization

#### 3.1.1 Chemical composition

The chemical composition of H-Y(80) zeolite was obtained by ED-XRF is shown in Table 2. It can be verified that the zeolite is basically composed of Si and Al, as expected, which enhances the high purity of this material. Additionally, the Si/Al ratio obtained was 32.91 This value is in accordance with the data reported by the Zeolyst International.^45^

**Table 2:** Elemental chemical composition for H-Y(80) as employed in reactions obtained from ED-XRF

Chemical composition (%)	H-Y(80) zeolite						
	Si	Al	Ca	Ti	Zr	Cu	Si/Al ratio
	96.33	2.93	0.46	0.16	0.05	0.06	32.91

#### 3.1.2 Thermal stability

**Fig. 1:**
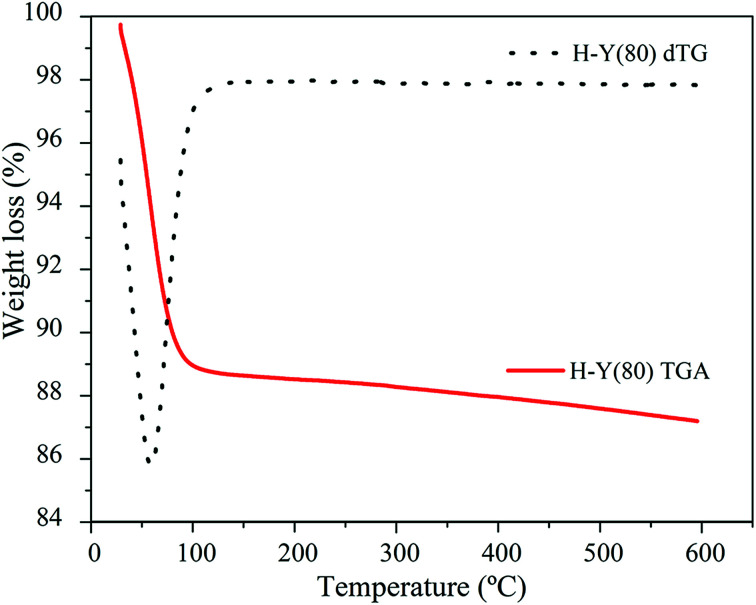
Thermogravimetric Analysis (TGA) and Derivative Thermogravimetric Analysis (dTG) of the H-Y(80) zeolite sample obtained with the flowing conditions: N_2_ flow 20 mL min^−1^ and heating rate of 10 °C min^−1^ from 40 to 600 °C.

The thermogravimetric profile obtained for the H-Y(80) zeolite is shown in the Fig. 1. The water molecules desorption occurred up to about 150 °C. The presence of impurities in the catalyst used was not observed.

#### 3.1.3 Structural characterization

**Fig. 2:**
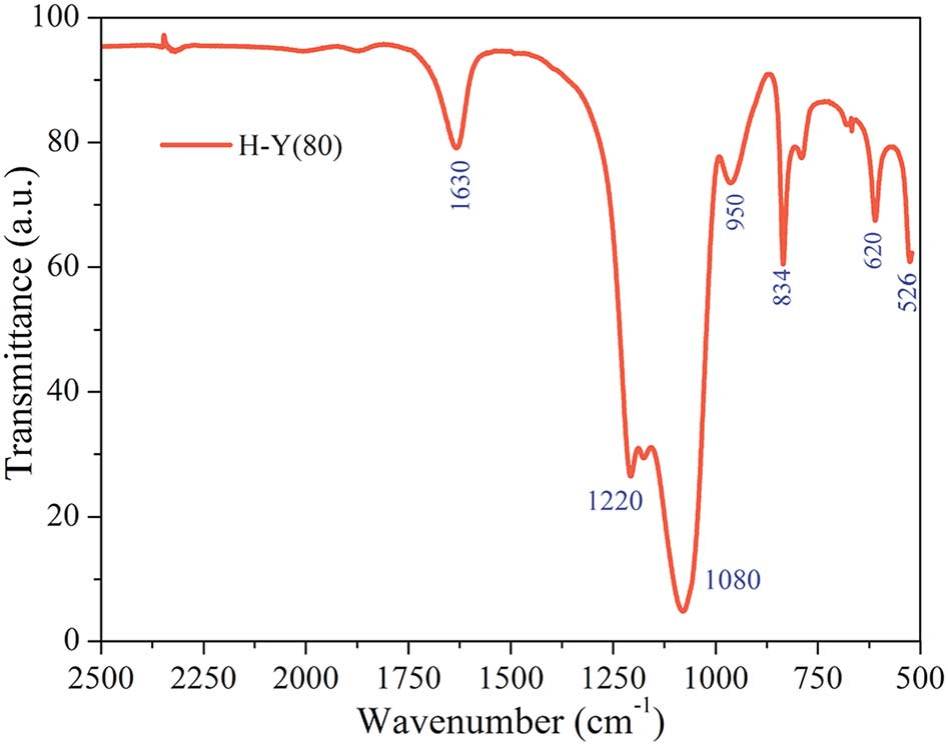
FTIR spectra of H-Y(80) from 2500 to 500 cm^−1^.

The FTIR spectra obtained can be seen in Fig. 2. Bands in the region of 1630 cm^−1^ refer to the presence of water molecules; the bands at 526, 620, 834 and 950 cm^−1^ are coupled to the Y zeolite formation structure, whereas the bands at 1080 and 1220 cm^−1^ are common to other zeolites.^49^

**Fig. 3:**
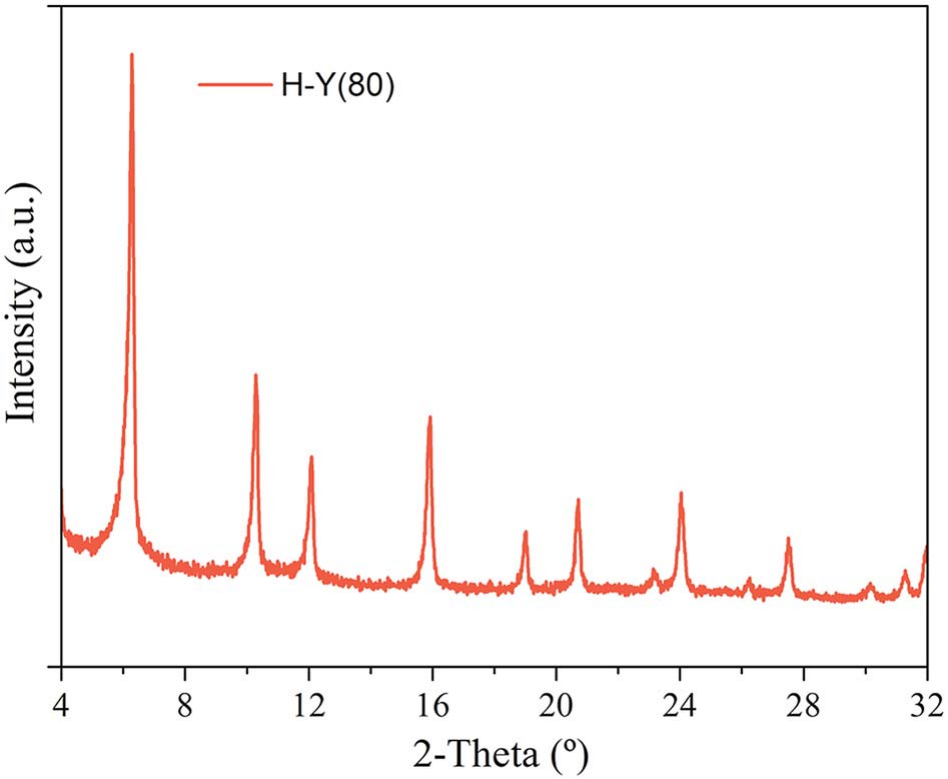
XRD patterns for H-Y(80) zeolite.

The XRD measurements are reported in the Fig. 3. The presence of crystalline impurities in the zeolite was not verified and the obtained diffractograms were consistent with the data reported in the literature.^50^ The diffraction peaks at 6.33, 10.34, 15.97 and 24.13° 2-theta confirm characteristic of the FAU topology.^50^

#### 3.1.4 Acidity measurements

**Fig. 4:**
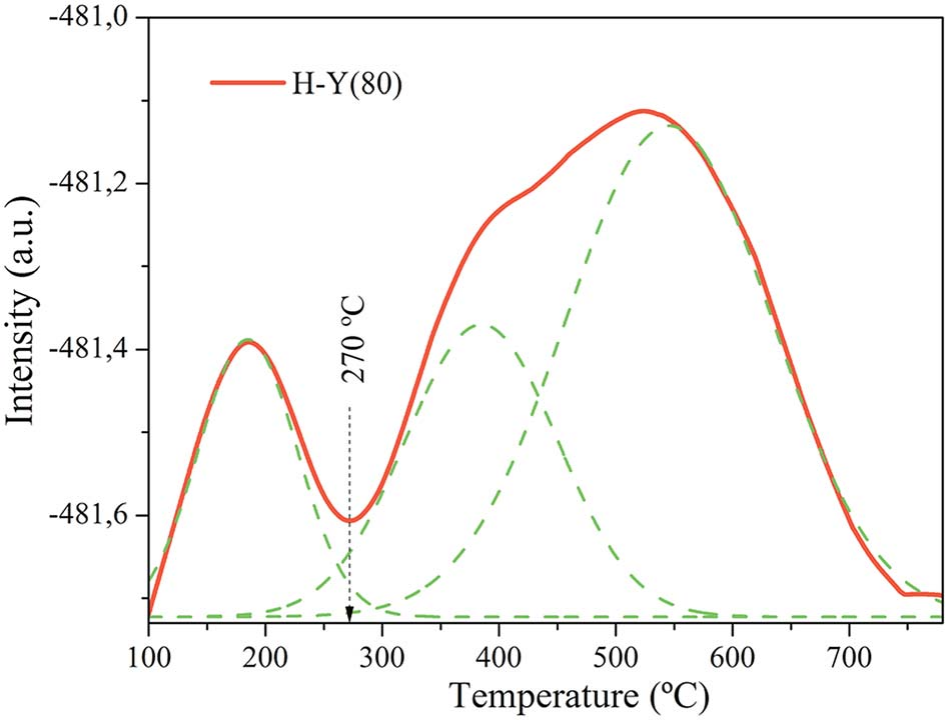
NH_3_-TPD thermograms at *β* = 10 °C min^−1^ for H-Y(80) zeolite.

The NH_3_-TPD profile obtained for the H-Y(80) zeolite is showed in the Fig. 4.

The specifics of the TPD curve, such as the temperature of the maximum desorption rate (*T*_max_), depends on the nature of the solid sample and also on the experimental conditions; consequently *T*_max_ values are not universally comparable quantities.^51^

In the NH_3_-TPD profile obtained at a *β* = 10 °C min^−1^ temperature ramp, three distinct desorption peaks corresponding to the weak, medium and strong acid sites, are observed respectively.^52^

To quantify weak and strong acidities, each NH_3_-TPD profile was separated into two sections with the “valley” between the two partially overlapped peaks used as a separation reference point. A vertical line was drawn between the “valley” and the baseline as presented in Fig. 4.^53^ The measured acid values are shown in Table 3.

**Table 3:** Measured acidity for H-Y(80) from NH_3_-TPD

Catalyst	*T*_peak_ (°C)		Measured acidity (μmol NH_3_ g^−1^)				
	LT-peak	HT-peak	Weak	Medium	Strong	Total	Weak acidity/(medium + strong acidity)
H-Y(80)	185	523	109.17	177.54	405.12	691.83	0.71

The amount of desorbed ammonia provides indicators of the acidity of the catalyst.^54^ Higher values indicate more active sites in the material. In this study, the zeolite H-Y(80) presented total acidity of 691.83 μmol NH_3_ g^−1^, which confirms the acid character of the catalyst. For another zeolites, similar results were reported by ref. 54–59. Additionally, in the studies carried out by Al-Dughaither & Lasa^53^ with H-ZSM-5 zeolites the authors observed an increase in total acidity according to the decrease of the Si/Al ratio.

Finally, although the zeolite presented weak, medium and strong acidity, according mentioned by Bakare *et al.*^60^ complete information about the zeolite acidity cannot be completely captured by NH_3_-TPD, since it does not distinguish the type of acid sites (Brønsted or Lewis). In this way, the understanding of the acidity becomes clearer after the Pyridine Fourier Transform Infrared Spectroscopy (Pyridine-FTIR).^60^

**Fig. 5:**
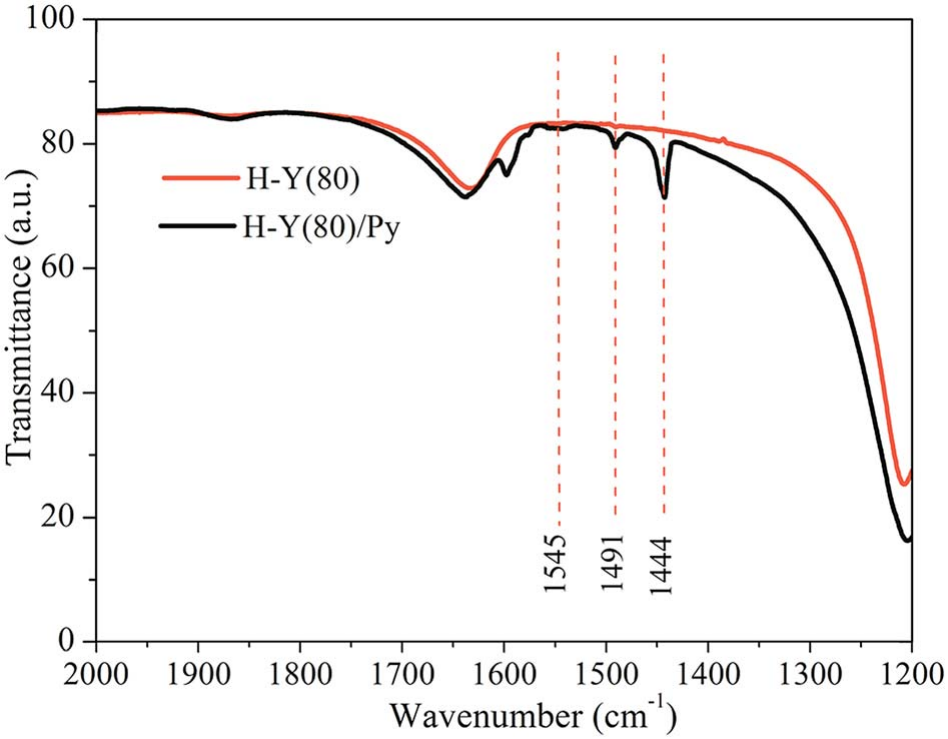
FTIR spectra for H-Y(80) zeolite with adsorbed pyridine molecules.

The FTIR spectra obtained for H-Y(80) zeolite with adsorbed pyridine are shown in Fig. 5.

In the zeolite FTIR spectra with adsorbed pyridine, the bands observed for the region of 1610 and 1640 cm^−1^ are attributed to the vibrational stretch of the *v*(–C=C) bond of the carbonic chain of the pyridine molecule.^61^ The bands in the 1444 cm^−1^ region are assigned to the pyridine molecules interacting by coordination with the Lewis acid sites.^62^

The bands in the region of 1545 cm^−1^ are assigned to the interaction of pyridine to the Bronsted acid sites resulting the formation of pyridinium ions from the protonation of Brønsted acid sites,^61^ whereas the bands in the 1491 cm^−1^ region refer to the interaction of pyridine on both active sites.^63^ Finally, the presence of the active sites show that the H-Y(80) zeolite sample used is acidic and corroborate with the results of the evaluation of its catalytic activity.

#### 3.1.5 N_2_ isotherms

**Fig. 6:**
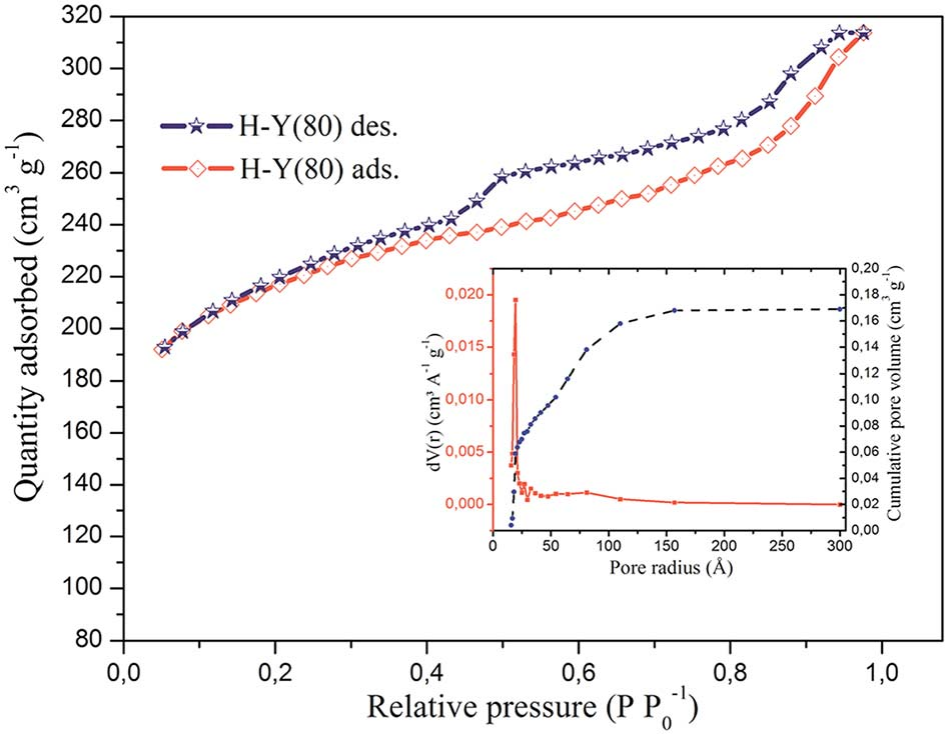
N_2_ adsorption–desorption isotherms and the pore size distribution of the H-Y(80) zeolite.

The N_2_ isotherms for the H-Y(80) zeolite are showed in the Fig. 6.

This zeolite presents an isotherm that resembles type IV, confirmed by the higher hysteresis at *P*/*P*_0_ = 0.45, where it is observed that the adsorption/desorption process is irreversible, resulting from the adsorption of N_2_ and capillary condensation in the mesopores.^64^ This indicates that in addition to the presence of micropores in this zeolite occurs the presence of mesopores. The calculated values for the surface area, pore volume and pore size are shown in Table 4.

**Table 4:** Textural properties of the zeolite samples obtained from of N_2_ physisorption at 77 K

Zeolite sample	Surface area (m^2^ g^−1^)			Pore volume^b^ (cm^3^ g^−1^)		Pore radius^c^ (Å)	
	*S*_BET-single-point_ a	*S*_BET-multi-point_ a	*S*_DFT_ b	*V*_BJH-adsorption_ c	*V*_BJH-desorption_ c	Adsorption	Desorption
H-Y	689.4	686.6	924.6	0.160	0.169	15.55	19.47

a Values obtained by BET method.

b Calculated with DFT method.

c Calculated with BJH method.

The values for the surface area obtained by the BET single-point and multi-point method, together with the specific area calculated by the DFT method, indicate that this zeolite has a high surface area, which is characteristic of these materials and consistent with the values reported by the manufacturer.^45^ The highest pore volume obtained by the BJH observed for Y zeolite was 0.169 cm^3^ g^−1^ on desorption.

### 3.2 FFA esterification pre-treatment

#### 3.2.1 Effect of molar ratio

**Fig. 7:**
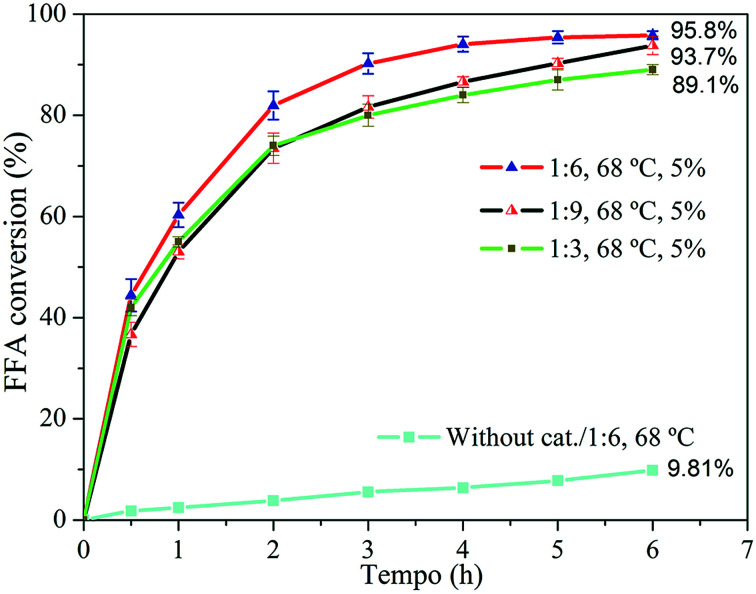
FFA conversion at 68 °C with oleic acid/methanol molar ratios of 1 : 3, 1 : 6 and 1 : 9 and reaction without catalyst at 1 : 6 and 68 °C for 6 h.

In general, the H-Y(80) zeolite was active in the FFA esterification pre-treatment. The influence of alcohol excess over FFA conversion is shown in Fig. 7.

The excess alcohol employed aimed to shift the reaction equilibrium towards the formation of the products, because the esterification is a reversible process. The FFA conversion was favored when the excess alcohol in the reaction medium increased from 1 : 3 to 1 : 6. Approximately 95% conversion was obtained after 6 h of reaction at 68 °C using 5% H-Y(80) zeolite. However, inhibition of the conversion was observed when the reaction was carried using molar ratio of 1 : 9, resulting in lower conversion values *versus* the ratio of 1 : 6 for the whole period. In this context, Kirumakki and coworkers^65^ studied the mechanism of the esterification reaction catalyzed by acidic zeolite and observed that, depending on the conditions, excess alcohol can block access to zeolite active sites, blocking the occurrence of adsorption of the fatty acid molecules, resulting in lower conversion since the acid adsorption is necessary for the reaction to occur. In this study, the most favorable molar ratio observed was 1 : 6.

#### 3.2.2 Effect of temperature

**Fig. 8:**
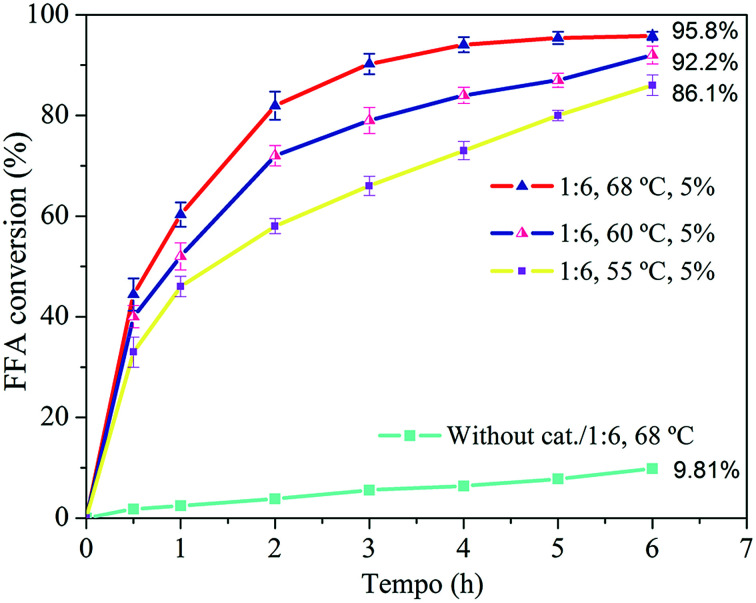
FFA conversion for temperatures of 55, 60 and 68 °C.

In the most favorable molar ratio, the effect of the temperature and catalyst amount was evaluated. The effect of the temperature can be seen in Fig. 8.

The uncatalyzed reaction that was carried out resulted in a conversion of 9.81% after 6 h of reaction. This result serves as the basis for the conditions which the zeolite is added to the medium and evidence the fact that the esterification can be also catalyzed by temperature. The temperature favored the reaction, conversion of FFA was higher for the condition where the temperature was higher, resulting in the conversion increase from 92.2 to 95.8% after 6 h. This is related to the higher probability of collision between the molecules, which favors the reaction. In addition, higher temperatures favor mass transfer through inside of the catalytic material structure.^66^

#### 3.2.3 Effect of catalyst amount

**Fig. 9:**
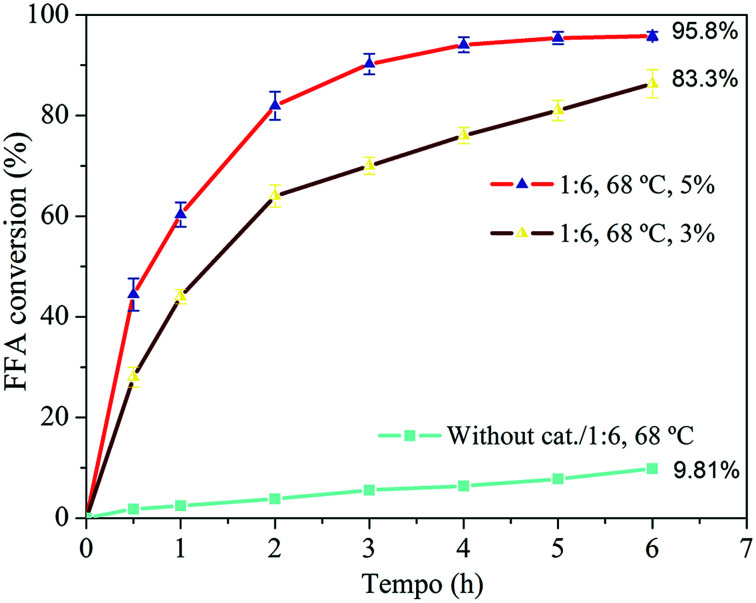
FFA conversion with 3 and 5% catalyst amount.

The effect of the catalyst amount is shown in Fig. 9.

The amount of catalyst directly influenced the reaction. The conversion increased from 83.3% to 95.8% after 6 h of reaction when increasing the amount of catalyst from 3 to 5%. The reaction was faster because of the greater number of active sites available to catalyze the reaction,^67^ which is a determining factor for the reaction.

The mechanism of esterification reaction catalyzed by acid zeolites has already been discussed by other authors,^41,68,69^ so will not be repeated here. The pore size of the H-Y(80) zeolite which is approximately 7.4 Å,^40^ makes it selective for the esterification of oleic acid, since in order for the reaction to take place it is necessary that the molecules of reagents and products can access the channel structure of the material. In this case, the simultaneous transesterification and esterification does not occur because the triacylglyceride molecules (20–30 Å) are larger than the zeolite pore size, making it selective for esterification of FFA.

The temperature of 68 °C, molar ratio of 1 : 6 and 5% of zeolite was obtained as the best condition. Under these conditions, conversion of oleic acid above 80% after 2 h of reaction as well as approximately 95% after 6 h of reaction was observed. These results show that the H-Y(80) zeolite is active for FFA esterification. This result suggest the potential application of this catalyst in processing of real waste grease.

Faujasite zeolite has been studied as a catalyst for the esterification reaction.^41,48^ The catalytic activity of the zeolites is not clearly related to the amount of active sites on the surface of the catalyst, but it is more dependent on the porosity and hydrophobic character of the materials.^41^

#### 3.2.4 Recyclability

**Fig. 10:**
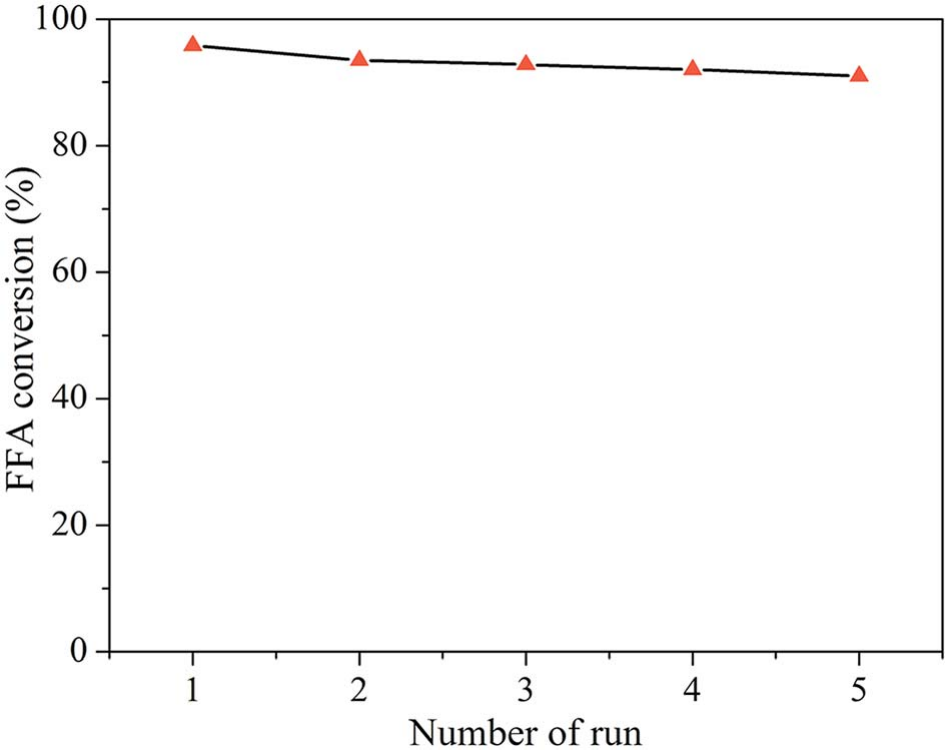
FFA conversion for five successive reaction cycles with H-Y(80) under the best conditions.

The catalytic activity of the zeolite H-Y(80) for sequential reaction cycles was evaluated by performing multiple catalyst reutilization runs under optimum reaction conditions (Fig. 10).

No significant drop in catalytic activity was observed although the catalyst was not regenerated at high temperature. Similar results for the recycling of zeolite catalysts were reported by Prinsen and coworkers.^41^ These recyclability results are superior to those observed in the literature for other solid acids.^70,71^ When the use of solid acids as catalysts for the biodiesel synthesis in several consecutive reaction runs, some problems such as the deactivation of the same ones, decrease of the catalytic activity and loss of structural characteristics are commonly observed.^27,71–73^

Finally, such results suggest that the H-Y(80) zeolite can be applied as a catalyst in a pre-treatment esterification of raw materials with high acidity (high FFA content). The conversion of 95% of the FFA after 6 h reduces the acidity of the reaction medium to insignificant values, allowing a possible biodiesel production process from low grade feedstocks with high acidity can be implemented using of a zeolite catalyzed esterification pre-treatment, followed by an alkaline transesterification without soap formation in the products or reduction in reaction yield.

## 4. Conclusion

The H-Y(80) zeolite presented acidity and has catalytic activity for the alkyl esters synthesis by esterification reaction. Under the best conditions (68 °C, 1 : 6 molar ratio and 5% catalyst amount), conversion of FFA around 95% was observed after 6 h reaction time. Results indicate that the concentration of FFA in the reaction medium was reduced to insignificant values and, therefore, the esterification pre-treatment catalyzed by the zeolite H-Y(80) was effective because it allowed that low grade feedstocks can be pre-treated. Moreover, these results suggest that the synthesis of biodiesel from raw materials with high acidity can be carried out using a zeolite-catalyzed pre-treatment esterification. Such, together with the easy catalyst separation from the reaction medium after the reaction, and the possibility of their reuse in new reaction cycles makes it advantageous the use of H-Y(80) zeolite in the FFA esterification pre-treatment.

## Conflicts of interest

There are no conflicts to declare.

## Supplementary Material
